# A systematic literature survey on recent trends in stock market prediction

**DOI:** 10.7717/peerj-cs.1700

**Published:** 2024-01-31

**Authors:** Prakash Balasubramanian, Chinthan P., Saleena Badarudeen, Harini Sriraman

**Affiliations:** 1 School of Computer Science and Engineering, Vellore Institute of Technology, Chennai, Tamil Nadu, India; 2 School of Mechanical Engineering, Vellore Institute of Technology, Chennai, Tamil Nadu, India

**Keywords:** Machine learning, Deep learning, Stock market prediction, Artificial intelligence

## Abstract

Prediction of the stock market is a challenging and time-consuming process. In recent times, various research analysts and organizations have used different tools and techniques to analyze and predict stock price movements. During the early days, investors mainly depend on technical indicators and fundamental parameters for short-term and long-term predictions, whereas nowadays many researchers started adopting artificial intelligence-based methodologies to predict stock price movements. In this article, an exhaustive literature study has been carried out to understand multiple techniques employed for prediction in the field of the financial market. As part of this study, more than hundreds of research articles focused on global indices and stock prices were collected and analyzed from multiple sources. Further, this study helps the researchers and investors to make a collective decision and choose the appropriate model for better profit and investment based on local and global market conditions.

## Introduction

Researchers from a range of disciplines are conducting studies on stock market forecasting. Many financial experts have attempted to address the problem of forecasting the upward and downward movements of the stock market, but have had only sporadic success. It is now more feasible than ever before to do so with the technology’s rapid advancements in computation capability, storage capacity, and algorithm accuracy. For stock market forecasting, researchers have experimented with a variety of strategies, algorithms, and attribute combinations. The characteristic of a prediction model depends on market-specific variables. The stock market plays a critical role to the rapid economic expansion of developing countries such as India. If the stock market falls, it will impact the growth of the country results in negative economic growth (Pillai & Al-Malkawi, 2018). There is a common misperception regarding the stock market is that purchasing and selling shares is a kind of gambling. Due to this reason, majority of people hesitate to take part in investments.

In earlier days, stock market predictions were majorly based on technical analysis and fundamental analysis. Technical analysis is used to predict the price movement of the stocks on short term basis. Candlesticks and chart patterns were predominantly utilized for technical analysis. There are many technical indicators like simple moving average (SMA), Bollinger band, Relative Strength Index (RSI), super trend *etc*., were exists but almost all these were considered as lagging indicators. Also, technical analysis does not take into account the fundamental aspects of equity’s key financial record. This is intuitively beneficial for making short-term investing decisions. Fundamental analysis majorly focused on the growth potential of the companies such as earnings, balance sheets, and revenues and so on. The investors compare the present growth of the company with the past earnings data and take their investment decision based on that. Fundamental analysis requires large amount of time and effort to analyze the past data due to which, fundamental analysis becomes least preferred choice among investors.

In recent times, algorithmic and artificial intelligence (AI) based approaches become popular and widely accepted among the analyst for predicting the trend of stock market. In particular, several machine learning models and neural networks have been mainly utilized for stock market prediction due to their specific characteristics such as non-linearity, time series nature of data, no assumptions, and a data-driven approach. The fundamental idea behind the deep learning technique is to do computations using neural networks. Long-short-term memory (LSTM) is a form of recurrent neural network (RNN) developed to address the challenge of long-term dependency (Althelaya, El-Alfy & Mohammed, 2018). The most renowned and promising method includes the deployment of artificial neural network (ANN), and RNN, which are essentially machine learning implementations. Machine learning entails artificial intelligence, which enables the platform to improve and learn from prior experiences without having to be performed repeatedly (Budhani, Jha & Budhani, 2012). Conventional machine learning prediction approaches include algorithms such as backward propagation, often known as back propagation losses and many researchers are now employing more collective learning approaches. It would forecast future highs with modest price and time delays, but another framework would forecast future highs using delayed highs.

Support vector machine (SVM) is a revolutionary neural network model that offers a potential result to the time series analysis. In contrast to so many classic neural networks that use the empirical risk minimization (ERM) concept, SVM uses structural risk minimization (SRM) principle, which seeks to lower the absolute limit of generalization infraction instead of training error (Yun, Yoon & Won, 2021). Following this hypothesis, the generalization deviation is constrained by the sum of both the training error and a confidence interval component which is dependent on the Vapnik-Chervonenkis (VC) dimension. This in turn makes SVM outperform other neural networks in terms of generalization performance, in accordance with this analysis. While comparable to classic neural networks, the effective use of SVM is dependent on the modeled data that having a certain degree of regularity. As a result, a basic SVM model would not be suitable for unstructured and complex time series financial data with changing dynamics. The modified SVM with a self-organizing feature map shows better results and outperforms simple SVM model in terms of prediction performance and convergence speed.

Even today with the availability and support of highly sophisticated tools and resources, forecasting the stock market is still challenging and difficult process owing to its uniqueness, non-linearity, high data rates, and susceptible local and global economic factors (Maini & Govinda, 2017). Wavelet neural systems have been implemented in recent decades, blending the advantages of neural networks and wavelet transforms, such as neural network dynamical estimation, institutionalization learning, simple structure, and so on, to make it much more successful in connecting the stock price correlation. Cuckoo Search—WNN combines the benefits of dynamic systems and artificial intelligence capabilities to prevent structural design impairment and faults of easily dropping into the local optimal solution, enabling it to embrace high-frequency information with greater precision, and function in under time with a simple structure (Yang & Suash, 2009). WNN, on the other hand, suffers from the limitations of the starting value.

With the advancement of technology, now-a-days investors are increasingly moving towards automated trading platforms known as algorithmic trading. Algorithmic trading in contrast with discretionary approach helps the analyst to quickly make wiser investing decisions. It may appear that matching the knowledge and integrity of an accomplished analyst who has been involved in the business for decades is an impossible endeavor, but despite the quantity of data accessible and digital transformations, it is quite feasible to develop algorithms that anticipate financial markets. Another method known as sentimental analysis is used for forecasting equity prices based on sentiments on social media feeds or news items, which aid in estimating the overall trend that a certain company’s or industry’s stocks may take focused on a collective opinion (Zhao et al., 2018). Digital networks have now grown into a mirror that depicts people’s reactions to any particular incident or piece of news. Any positive or negative public perception of a business organization may have an influence on its stock value. To anticipate the stock market prices of numerous companies using sentiment analysis on social media data, including tweets regarding the company in question. People share their ideas and views about a certain issue, such as news, movies, events, and comments linked to products, *via* social networking such as Facebook, Twitter, *etc*. Business analysts and leading investment banks may utilize this information from social networks to get consumer input on their products and use it to enhance their planning, management, and product development strategies. The opinions and comments of users are extracted using sentiment analysis, which categorizes them as positive, negative, and natural sentiment. Though sentiment analysis has been given many different understandings in the literature, in simplest words, it is a method for extracting meaningful content based on an individual’s opinion from unprocessed internet data. The stock’s behavior over time is significantly influenced by news feeds as part of qualitative research. This further demonstrates the close relationship between media and stock market trends (Mohan et al., 2019; Chen & Chang, 2010; Chen & Tanuwijaya, 2011). Twitter is undoubtedly the fastest and most trustworthy means to consume information, it can be asserted with certainty. Like Twitter, Yahoo Finance API is also utilized to fetch the data directly from exchanges and used it for train and tests the algorithm and provides predictions.

The main objective of this study is to provide an understanding of research techniques and methodologies presently applied in the field of stock market prediction and analysis. This study helps the researchers, investment analyst, and market participants to a greater extent to choose the appropriate methods to predict the stock price movement so that they can take a better financial decision based on the suggestion provided by the chosen technique.

This article is formulated as: “Introduction” provides a detailed introduction to stock market forecasting, “Research Method” focused on research methods and techniques used in stock market analysis, “Challenges and Discussion” details various challenges and discussion, “Conclusions” concludes the article, and finally “Future Scope” provides future direction.

## Research method

In the past, there are various techniques used among researchers to predict stock market price movements. Whereas in recent times, in addition to existing technical indicators and fundamental attributes, artificial intelligence (AI) based study becomes popular and widely accepted to predict stock price movements. The researchers and analysts employed different machine learning and deep learning models, neural networks, fuzzy logic systems, and sentimental analysis to understand the stock market price movements. There are many research articles, white articles, investment-related blogs, and websites available to assist investors to make wise decisions about their investment decisions.

The main purpose of this research study is to find empirical evidence in the field of stock market analysis and predictions through existing literature study and statistical data. In this process, five key research questions (RQ1 through RQ5) are considered and this study helps to find the answers to those questions with the help of an exhaustive literature study. A total of over 300 research articles from various multiple sources such as Scopus database, Google scholar, Science Direct, IEEE, and Web of Science were considered. In addition, other formats of input data from social media handle such as Twitter and Facebook, and various authenticated financial websites like money control, Bloomberg, *etc*., were also been identified for the analysis.

The research questions formulated as part of this study are as follows:

**RQ1:** What are the research techniques or methodologies employed in recent times to predict stock market movements?

**RQ2:** What are the different sources of datasets considered for the stock market predictions?

**RQ3:** What are the most popular journal publishers available in the domain of stock market investments?

**RQ4:** What countries show research interests in equity/capital market investments?

**RQ5:** What is the most popular evaluation metrics used in a stock market analysis?

### Stock market prediction using machine learning techniques

Usmani et al. (2016) used a combination of attributes and Artificial Neural Networks to foresee the stock market volatility of KSE. This study utilizes artificial neural networks such as single and multi-layer perceptron, radial base function (RBF), and SVM for their analysis. This study is based on parameters such as commodity prices, gold and silver prices, market history, news, global currency rates, *etc*. The news and Twitter feed were given as inputs and were processed to give the outcome as positive and negative. This study concludes that the multi-Layer perceptron algorithm outperformed the other algorithms and also derived that petrol price played the most significant role in the evaluation of the performance of KSE and that the foreign exchange had no effect on the KSE performance.

A survey has been conducted on efficient regression models in predicting stock market prices based on historical data by Sharma, Bhuriya & Singh (2017). Different regression techniques like polynomial regression, RBF regression, sigmoid regression, and logistic regression (LR) were selected for the survey. It is concluded that a higher range of variables might improve the multiple regression analysis.

Kumar et al. (2018) performed stock price prediction with supervised learning procedures like SVM, KNN, Naïve Bayes, random forest (RF), and SoftMax. In this study several technical indicators were integrated with machine learning models. Moving averages for 10 and 50 days were evaluated for feature extraction, the Relative Strength Index (RSI), which indicates if the stock is overvalued or oversold, the rate of change (RoC) to measure the price change from one timeframe to the next, volatility to indicate the scattering of returns for a given firm, the Disparity Index (DI) to measure the relative strength of a selected moving average to the most recent closing price, the stochastic oscillator to outline the position of the trading session to the relative high-low range, momentum indicator Williams % R to outline the level of final closing price relative to the highest point, and volume price trend and Commodity Channel Index (CCI) are calculated to determine the current price level in relation to the median price over a specific time period. The analysis was performed on the past 5–10 years of historical data for Amazon, Bata, Bosch, Cipla, and Eicher motor. The performance of the models was appraised based on evaluation metrics like Accuracy, precision, recall and F-measure. The analysis indicates that for huge data sets, such as Amazon, Bata, and Bosch, the RF topped the other models with respect to accuracy, but for smaller datasets, such as Cipla and Eicher, the Naïve Bayes approach produced the greatest performance in terms of accuracy. The study concludes that by limiting the number of statistical features, the efficiency of the algorithms in predicting stock market movement decreases.

A similar organized literature survey was conducted by Mankar et al. (2018) along with his team, through social sentiments from Twitter. Naïve Bayes and SVM were selected for this classification examination. As part of preprocessing, this study applies Python’s Natural Language Toolkit (NLTK) to compute conditional recurrence and characteristic frequency. It was determined that SVM was the most efficient and viable method for anticipating stock market movements using social sentiments.

A framework is proposed by Sadia et al. (2019) to predict the stock market prices based on historical data. This study utilizes an RF classifier, SVM classifier, and RF algorithm for their analysis. In addition, a confusion matrix has been constructed for the assessment of the models’ performance. Upon measuring the accuracy, it was concluded that the RF algorithm is most suitable for the stock market prediction based on various data points from the historical stock data. Kompella, Chilukuri & Kalyana (2019) evaluates the effectiveness of random forest for predicting stock prices with logistic regression based on sentiment analysis. Historical stock data and news headings are given as inputs. The polarity score is calculated using sentiment analysis. Further, several error metrics such as variance score, Mean Absolute Error (MAE), Mean Squared Error (MSE), and Mean Squared Log Error (MSLE) was used to quantify the effectiveness of the algorithm. It is ascertained that the RF algorithm outperforms logistic regression for forecasting the stock market on sentiment classification.

To forecast the equities listed on the NSE and NYSE, Hiransha et al. (2018) employed deep learning models. This study found that neural network models excelled linear models, in particular ARIMA, and was based on the forecast of five stock prices mentioned in the two indexes. Five NYSE-listed big capitalization stocks were chosen for the Vijh et al. (2020), study’s (Vijh et al., 2020) closing price prediction analysis. They employed machine learning approaches such as ANN and RF for their analysis. The performance of the models was reviewed using assessment instruments including mean absolute percentage error (MAPE) and root mean square error (RMSE). According to the results obtained, ANN outperformed RF in terms of stock value prediction accuracy.

In order to forecast a stock market’s future trend based on specific external contributing variables like news and social media posts, Khan et al. (2020) established a framework. According to the study findings, the accuracy of stock forecasts is positively impacted by pre-processing stages like the elimination of spam tweets and feature selection. Rao, Srinivas & Mohan (2020) conducted another study to analyze stock movement based on comparing several methodologies with their benefits and limitations. The evaluation and comparison of eight supervised machine learning models were used for forecasting the stocks in the Nifty 50 index (Singh, 2022). Based on historical data from the previous 25 years, the study was carried out. The study demonstrated that linear regression outperformed neural networks because it handles linear dependence data better than SVM and gradient descent. By using a linear regression model with three-month moving averages and exponential smoothing forecasts, the NYSE stock price movements were examined (Umer, Awais & Muzammul, 2019). The outcome demonstrated that forecasts using exponential smoothing outperformed those using linear regression and three-month moving averages.

A review of stock price movement was conducted by Soni, Tewari & Krishnan (2022) by examining numerous machine learning algorithms. For the comparison study, the various types of methodologies, including standard machine learning (ML) techniques, deep learning models, neural networks, time series analysis, and graph-based approaches, were chosen. Rouf et al. (2021) undertook a further comparison of stock market forecasts based on research done in the previous 10 years. The types of data used as input, various pre-processing techniques, the machine learning and deep learning models used for predictions were all taken into consideration throughout the analysis. SVM is the better performing machine learning model for stock market analysis, according to the study’s findings. Additionally, other approaches, such as DNN and ANN, offer quicker and more precise projections of stock prices.

An analysis of the use of several ML algorithms useful in forecasting the future values of equities in the financial sectors has been performed (Obthong et al., 2020). For the purpose of forecasting stock price fluctuations, Parmar et al. (2018) constructed two models: regression and LSTM. According to the results, LSTM outperformed regression in terms of prediction accuracy. Similar to this, Pathak & Pathak (2020) examined four machine learning models for stock market prediction: RF, SVM, KNN, and LR. The study’s findings indicated that random forest outperforms the other algorithms in terms of accuracy, precision, sensitivity (recall), and F-score (F1-score). Lokesh et al. (2018) combined sentiment analysis and machine learning algorithms to determine the trend of a particular stock. In addition, based on the derived results, the risk exposure towards the particular company has been determined and notified to the user. Mehta, Pandya & Kotecha (2021) integrated sentiment analysis with deep learning models to enhance the prediction accuracy of the stock market. The results indicate that the combination of deep learning models along with sentiment analysis has a positive impact on stock price movement predictions.

Further on the topic of stock market analysis, Strader et al. (2020) performed a systematic literature review (SLR) on four categories: artificial neural networks, support vector machines, genetic algorithms, and other hybrid approaches. This study comes to the conclusion that hybrid approaches are useful in overcoming the shortcomings of a single method, genetic algorithms are used to suggest suitable stocks for a portfolio, artificial neural networks are suitable for stock value index predictions, SVM is useful for forecasting overall trend of indices, and genetic algorithms are used to suggest the suitable stocks in a portfolio. The research articles on stock market forecasting using machine learning approaches are described in Table 1.

**Table 1:** Stock market prediction using ML techniques.

Authors	Scope	Input features	Feature extraction	Prediction algorithm
Usmani et al. (2016)	KSE	Price data	Normalization	SLP/MLP/RBF/SVM
Sharma, Bhuriya & Singh (2017)	Global	Price data	NA	PR/RBF/Sigmoid/LR regression
Kumar et al. (2018)	NSE	Price data	Technical indicators	SVM/RF/KNN/NB/SoftMax
Mankar et al. (2018)	Global	Tweet text	Chi square test	NB/SVM
Sadia et al. (2019)	Global	Price data	Scaled raw data	RF classifier/SVM classifier/SVM
Kompella, Chilukuri & Kalyana (2019)	Global	Price Data & News	Smoothing (polarity score)	RF algorithm
Hiransha et al. (2018)	NSE & NYSE	Price data	Normalization	MLP/RNN/LSTM/CNN
Vijh et al. (2020)	NSE	Price data	Technical indicators	ANN/RF
Khan et al. (2020)	KSE, LSE, NASDAQ & NYSE	Price data, Tweet Text & news	Technical indicators	RF/ET/GBM
Rao, Srinivas & Mohan (2020)	Global	Price data	NA	Holt-Winters/ANN/HMM/ARIMA/RNN
Singh (2022)	NSE	Price data	Scaled raw data	ANN/LR/SGD/SVM/AdaBoost/RF/KNN/DT
Umer, Awais & Muzammul (2019)	NSE	Price data	Normalization	LR/3MMA/ES
Soni, Tewari & Krishnan (2022)	Global	Price data	Binary features	PLS Classifier/SMO/ExtRa/ LSTM/ CNN/ ARIMA/GAM using Fourier transformations
Rouf et al. (2021)	Global	Price data & Tweet text	Aspect based correlation	ANN/SVM/NB/GA/FA/DNN/RA/HA
Obthong et al. (2020)	Global	Price Data	NA	KMeans/SOM/RF/MLP/LSTM/RNN/GA/SVR/MCS/ANN/CART/GP/BSM/GRNN/RBF/BPNN/LR/HMM/SVM/KNN/LR
Parmar et al. (2018)	NSE	Price data	Normalization	Regression/LSTM
Pathak & Pathak (2020)	NSE	Price data	Normalization	RF/SVM/KNN/LR
Lokesh et al. (2018)	NSE	Price data, Tweet text	Toordinal feature extraction	Machine learning models
Mehta, Pandya & Kotecha (2021)	NSE	Price data, News	Polarity, Stemming	LSTM

### Stock market prediction using neural networks

Olivier (2007) provided an outline of the modeling method using ANN for anticipating stock market prices. This study also addressed the challenges experienced in using neural networks for predicting future stock market changes. A similar study was done by Honghai & Haifei (2012), in which they devised a two-stage neural network for stock market prediction by integrating SVM with empirical mode decomposition. The experimental findings suggest that the integrated model outperforms the simple SVM in terms of prediction performance. White (1988) forecasted the IBM daily common stock price using three layers of a feedforward neural network, one input, one hidden layer, and one output layer. The team employed a 5,000-day dataset to perform their analysis. The first 1,000 days of data were utilized for training, while the remaining days were used for testing. The neural network’s performance was unsatisfactory, but they offered useful information for integrating neural networks in forecasting the stock market.

A neural network model was used to forecast the closing level of the Indian S&P CNX Nifty 50 Index (Majumder & Hussian, 2008). The study analyzed 10-year data sets of the S&P CNX Nifty 50 Index final price from January 1, 2000, to December 31, 2009. Four of the 10 years of data were utilized for validation. The authors present an ideal ANN structure, which is a three-layer feedforward hybrid backpropagation neural network with ten input neurons, a hidden layer of five neurons, and one output neuron. In their forecasts, they had the best performance of 89.65% and the lowest precision of 69.72%.

The authors Mehrara et al. (2010) have used moving average indicator to compare the performance of MLP feedforward with backpropagation and group method of data handling (GDMH) with GA in forecasting the stock price index of Tehran Stock Exchange (TEPIX). The results revealed that GDMH with GA outperformed MLFF with a backpropagation network.

A comparative analysis on the Dow Jones Industrial Average utilizing three methods (MLP, adaptive neuro-fuzzy inference, and generic evolving and pruning RBF neural network) were performed in the study (Quah, 2007). The study examined 10 years of information from 1995 to 2004 for 1630 Dow Jones Industrial Average shares. The authors in this study (Mandziuk & Jaruszewicz, 2007) implemented a neuro-evolutionary neural network with GA to forecast the short-term stock index of GSE. The study data set included the GSE (DAX), TSE (NIKKEI 225), NYSE (DJIA), and EUR/USD and USD/JPY currency exchange for a 15 years period. Their findings revealed that the neuro-evolutionary technique outperformed alternative testing models. Other neural network architectures, in addition to the feedforward neural network, have been used in stock market prediction. RNN is another type of neural network design in which the network connections form a guided cycle. The result concluded RNN has several internal states that displays dynamic temporal patterns.

Schierholt & Dagli (1996) employed MLP and a probabilistic neural network to forecast the S&P 500 index. From February 1994 to September 1995, the data set included the daily closing S&P 500 index as well as foreign exchange rates for the Yen, Pound, and Mark. The results indicated that the probabilistic neural network outperformed the MLP.

Another study that achieves something similar is Charkha (2008), where they forecast both the pattern and validity of stock prices using a feed-forward neural network and a radial basis neural network with backpropagation. They retrieved data from the NSE since November 2005 and the study revealed that the feed-forward neural network with backpropagation is advantageous for trend prediction, with almost 100% accuracy as compared to the radial basis neural network’s 80% accuracy. Furthermore, the radial basis neural network outperformed the feed-forward neural network in stock price prediction, gaining a greater percentage of accuracy. In their stock market prediction in the trading study, Mizuno et al. (1998) used neural networks. They forecast the Tokyo stock market, and the approach for doing so is more accurate than 63% of genetic algorithms. The forecasting procedure for changes in the index stock market is handled by a combination of neural networks and genetic algorithms.

A performance comparison of ANN and SVM was carried out in 2011 by Kara, Acar Boyacioglu & Baykan (2011). These two classifiers received ten technical indications in order to forecast the movements of the National 100 Index of the ISE. Researchers discovered that ANNs’ predictive power is noticeably superior to SVM. Popular ANNs that can forecast both price movement direction and price value include feed-forward ANNs. The studies on stock market prediction utilizing neural networks are shown in Table 2.

**Table 2:** Stock market prediction using neural networks.

Authors	Scope	Input features	Feature extraction	Prediction algorithm
Olivier (2007)	Global	Price data	Normalization	ANN
Honghai & Haifei (2012)	SSE	Price data	Decomposed intrinsic mode functions (IMFs)	Combined SVM and EMD
White (1988)	IBM common stock	Price data	NA	Neural network modelling
Quah (2007)	NYSE & NASDAQ	Price data	Technical indicators	MLP/ANFIS & GGAP-RBF
Mandziuk & Jaruszewicz (2007)	GSE, NYSE & TSE	Price data	Technical indicators	Neural networks and GA
Schierholt & Dagli (1996)	NYSE	Price data	Normalization	Probabilistic neural network (Custom Model)
Charkha (2008)	NSE	Price data	Normalization	Feed forward network with back propagation/radial basis network
Mizuno et al. (1998)	TSE	Price data	Normalization	Custom neural network model
Kara, Acar Boyacioglu & Baykan (2011)	ISE	Price data	Technical indicators	ANN/SVM
Zhi et al. (2017)	Global	Price data	NA	CSWNN/WNN

Zhi et al. (2017) developed a network to foresee stock market activities and predict stock prices by optimizing the basic WNN parameters. To improve the initial parameters, this work uses a brand-new meta-heuristic technique called Cuckoo Search. The outcome of the trials demonstrates that in terms of the degree of fitting and prediction accuracy, CS-WNN is superior to WNN.

### Stock market prediction using sentiment analysis

Gao et al. (2022), looked into the sentiment analysis-based prediction and proposed a framework with varying weights to boost prediction performance of the model. In sentiment analysis, news articles’ sentiment scores were determined using the popular Loughran-McDonald sentiment dictionary. The sentiment index for each news source was then calculated by integrating those sentiment scores. The RNN was then used to establish a series of basis classifiers depending on the market data and sentiment indices from various news publishers, and the evidential reasoning rule was used to integrate these base classifiers to predict the movement of the stock market index.

Duan, Liu & Wang (2021) quantifies the Chinese stock industry’s reactivity related to novel coronavirus 2019 (COVID-19). Using 6.3 million textual data pieces acquired from the government publications media and Chinese Social media blog sites, they built two COVID-19 sentiment indexes that reflect the feelings associated with COVID-19. Their stock market sentiment indicators are real-time, forward-looking indexes. They discovered that COVID-19 attitudes predicted stock market volatility and pay scales accurately.

Based on the partial least squares technique, Gong et al. (2022) suggest a new investor sentiment index (NISI). In three different methods, their sentimental analysis performs better than many other sentiment indicators now in use. First, the in-sample results demonstrate that the NISI has a higher level of predictive ability than the others. While the NISI is also beneficial during times of crisis, most mood indicators only demonstrate predictability during non-crisis periods. Additionally, the NISI shows a more obvious advantage in predicting over longer time horizons. Second, additional research reveals that, in contrast to the others, the NISI shows strong predictability before and during moments of market turmoil in China. In contrast to most of the others, the NISI is still considered effective despite taking leverage impact into account. Lastly, out-of-sample research shows that the NISI outperforms other sentiment metrics.

Malawana & Rathnayaka (2020) used a machine learning approach to do analysis and data processing utilizing the Spark model on the Google cloud platform. Logistic regression and Naive Bayes were effective in categorizing emotional reactions. The study’s key finding was that public perception has a significant influence on how economic forces and economic variables such as rate of interest, public trust, and faith in the bond market operate. Monetarism, political changes, unanticipated pandemics, and interest rates are some of these influences. In Khatri & Srivastava (2016), sentimental analysis was done on the data that was taken from Stock Twits and Twitter. To determine the user’s comment’s mood, the data was evaluated. Four categories were used to group these comments: joyful, up, down, and rejected. An artificial neural network was given the polarity index and market data to forecast the outcomes.

Mehta, Malhar & Shankarmani (2021) concentrate on many approaches to studying the stock market’s patterns in real time. The approach with the highest reliability is the best and even more recommended method of projection. The authors used three distinct models for their work, as well as sentiment classification on tweets concerning the firm or commodity. The classification’s findings have provided clear and incisive insight into the market’s volatile movements as well as a fresh strategy for investors to use when deciding where to stake their capital. For every stock, the ARIMA model had the best accuracy.

Jing, Wu & Wang (2021) suggest a hybrid model for stock price prediction that combines a deep learning approach with a sentiment analysis model. They used CNN model to categorize the hidden sentiments of investors that they extract from a significant stock forum. Then, using the LSTM neural network technique to analyze the stock market’s technical indicators and the sentiment analysis findings from the first stage, they suggest a hybrid research model. In order to confirm the efficacy and applicability of the suggested model, this study has also carried out real-world tests from six important sectors across three-time intervals on the Shanghai Stock Exchange (SSE).

The current digital world has altered the way we conduct our business, owing primarily to web technologies such as big data analytics, cloud computing, and sentiment classification. Sentiment analysis, also known as opinion mining, uses text mining and natural language processing (NLP) to analyze user opinions, assessments, sentiments, and attitudes, and to discover and extract sensory knowledge through emotions. This (Bhardwaj et al., 2015) study investigated the importance of sentiment classification for stock market indices such as the Sensex and Nifty in forecasting stock prices on the Indian stock exchange.

Sentiment analysis was utilized by Rajendiran & Priyadarsini (2021) to compare several conventional stock market forecast algorithms. As a result, the reliability of the revised stock market categorization model was not increased. The survival analysis demonstrated that the prediction outcome was not enhanced by the emotional analysis approach. The performance of stock market forecasts was computed using a wide variety of validation using traditional methodologies. Finally, a variety of machine learning and classification techniques were to enhance stock market predictive ability with the highest level of accuracy in the shortest amount of time.

Owen & Oktariani (2020) assess the idea of utilizing past data and sentiment evaluations derived from microblog language data to improve stock market prediction accuracy. The sentiment score is extracted using an ensemble-based approach that leverages the capability of CNN, MLP, and LSTM. They provide the SENN model, which is trained by analyzing sentiment in text data from StockTwits microblogs and historical Boeing stock data. Furthermore, they offer a one-of-a-kind approach for assessing the performance of stock market forecasting, namely adjusted MAPE (AMAPE), a variant of the traditional mean absolute percentage error (MAPE) metric. Further, Table 3 summarizes the findings on sentiment analysis-based stock market forecasting.

**Table 3:** Stock market prediction using sentiment analysis.

Authors	Scope	Input features	Feature extraction	Prediction algorithm
Gao et al. (2022)	NYSE	Price data & Financial news	Sentiment polarity using LMD dictionary	RNN-ER-GA
Duan, Liu & Wang (2021)	SSE	Financial News & text	Scaling raw data	Naive Bayes/SVM/Xgboost
Gong et al. (2022)	SSE	Price data & Financial news	Technical Indicators	NISI
Malawana & Rathnayaka (2020)	CSE	Twitter Tweets	Tokenization/Removing Stop words/Symbols	Custom visual representation of sentiment time series
Khatri & Srivastava (2016)	NSE	Twitter & Stock Twits	Polarity index	ANN
Mehta, Malhar & Shankarmani (2021)	NSE	Twitter Tweet & Stock ticker	Regual expression extraction	LSTM/ARIMA/LR
Jing, Wu & Wang (2021)	SSE	Textual data & Technical Indicators	Optimal classification accuracy	LSTM/CNN
Bhardwaj et al. (2015)	NSE	Price data & Financial news	NA	Sentiment analysis for Stock Market prediction on the basis of variation in predicted values
Owen & Oktariani (2020)	NYSE	Financial news & microblog text data	Ensemble based model	CNN/LSTM/MLP/SENN

### Stock market prediction using other techniques

The MG-Conv, a multi-graph CNN-based index pattern forecast model, was proposed by Wang et al. (2022b). A one-dimensional convolutional neural network and data normalization were originally proposed to extract fundamental properties from previous transaction data. The terminology known as static and dynamic graphs were then coined for two types of correlation graphs. The results of multigraph convolution on these two graphs were then converted into predicted values using fully connected networks. A total of 42 Chinese stock market indices were utilized as experimental data. Traditional approaches such as LSTM, 3D-CNN, GC-CNN, and AD-GAT were utilized as comparison benchmarks. The results indicated the method's tenacity and capacity to reduce the average model complexity by 5.11%.

A nature-inspired algorithm that mimics the human ear’s auditory system by following its natural pathways called AA was proposed by Oyewola et al. (2021). To compare the performance of AA, high-performance machine evolutionary computation and prolonged stochastic processes are utilized. Machine learning approaches such as LR, SVM, feed forward neural network, and RNN were used in addition to continuous-time models such as the stochastic differential equation (SDE) and geometric brownian motion (GBM). The results indicate that AA beat the other algorithms evaluated in this study in terms of overall effectiveness since it significantly decreased prediction error to the bare minimum.

In order to forecast long-term stock trend behavior, Zhao & Wang (2015) introduced a unique data mining technique in their study. They proposed a novel outlier mining technique to discover abnormalities in the market index based on volume sequences of high-frequency data. Such anomalous deals deduce always from the stock price on the stock exchange. By utilizing the clustered structure of such deviations, their system correctly anticipates stock market volatility in the actual worldwide market. The results of their experiment demonstrated that, when used over a long period of time, their suggested strategy generates profits on the Chinese stock exchange.

In this study, Vlasenko et al. (2018) suggest a hybrid five-layer neuro-fuzzy model and an associated learning algorithm for time-series prediction tasks in the stock market. In order to improve computational speed and representational capabilities in processing highly non-linear volatile data, multidimensional Gaussian functions were utilized in place of polynomials in the fourth layer of the suggested model as opposed to the traditional ANFIS design.

By taking the parameters linked to COVID-19 into consideration, Jindal et al. (2021), proposed a study that seeks to improve the stock market forecast capacity of several popular prediction models. DT Regressor, RF Regressor, and SVR are the forecasting methods considered for their study. The United States, Russia, and India are the nations that are now most impacted by COVID-19. Therefore, they used mean absolute percentage error (MAPE) and root mean square error (RMSE) to analyze the performance of various prediction algorithms on the S&P 500, Nifty50, and RTS Index. The results reveal that when the COVID-19 characteristics are employed, all of the strategies tested performed better.

In this work, Umadevi et al. (2018) have made an effort to develop a stock exchange forecasting system that takes into account various equity-specific factors. The equity ratings were obtained, and then the analysis was performed. Throughout this study, the equity ratings are shown using a variety of graphs, and the time series model ARIMA (autoregressive moving average) is used to forecast the scores. The findings demonstrate that the time series model successfully predicted market ratings with a significant degree of accuracy. To determine how each element related to market performance, separate studies of each factor were conducted. Additionally, the findings suggest that machine learning techniques might be used to forecast market behavior.

Three fuzzy logic controllers are utilized in the algorithm by Lauguico et al. (2019) designed to implement a certain trading strategy. Trigger functions such as candlestick characteristics and Bollinger Bands (BB) were used to assess the effectiveness of the purchase, hold, and sell signals. A specific stock organization provided information on equity markets. The opening and closing prices utilized to help compute the BB are included in these figures. The raw and generated values are the crisp input parameters of the fuzzy inference system (FIS). The classifiers were classified into very low, low, high, and very high levels based on the entered default parameters used by traders. Fuzzy logic was utilized to create association rules that would offer signals indicating the effectiveness of an execution recommendation.

Even though there is a variety of data structures that can assist in identifying the proper clusters from a fuzzy model’s divine of reasoning, de Carvalho Tavares, Ferreira & Mendes (2022) proposed a unique fuzzy model based on a red-black tree (RBT) data structure in order to enhance the prospects of achieving improved projections. The RBT data structure, supports more balance, enabling more certainty. The suggested model performs superior predicting when compared to well-known fuzzy models in the literature.

In this study, Wang et al. (2022a) forecast the stock market index using Transformer, the most recent deep learning framework. The transformer was created to address the issue of natural language processing and is now used for time series forecasting. The transformer can more accurately represent the fundamental principles governing stock market movements because of its encoder-decoder design and multi-head attention mechanism. They perform a variety of side studies on the world’s leading equities, including the CSI 300, S&P 500, Hang Seng, and Nikkei 225 Index. Each of the research demonstrates that transformer outclasses other traditional strategies and can deliver financial support to investors. The studies on different methods of stock market forecasting are summarized in Table 4.

**Table 4:** Stock market prediction using other techniques.

Authors	Scope	Input features	Feature extraction	Prediction algorithm
Wang et al. (2022b)	SSE	Price data	Normalization	LSTM/3D-CNN/GC-CNN & AD-GAT
Oyewola et al. (2021)	NGX	Price data	Technical indicators	AA/LR/SVM/FFN/RNN/SDE & GBM
Zhao & Wang (2015)	SSE	Price data	Tick-by-tick data	Outlier mining algorithm/Cluster algorithm
Vlasenko et al. (2018)	Global	Price data	NA	MIMO neuro-fuzzy model with multidimensional Gaussian functions
Jindal et al. (2021)	NSE/NYSE/MOEX	Price data	Scaling raw data	DT/RF & SVR
Umadevi et al. (2018)	NYSE	Stock score	NA	ARIMA
de Carvalho Tavares, Ferreira & Mendes (2022)	B3/NYSE	Price data	Scaling raw data	Hybrid fuzzy time series model with red–black tree data structure
Wang et al. (2022a)	SSE/NYSE/HKEX/TSE	Price data	Normalization	RNN/CNN/LSTM/Transformer
Sharma & Juneja (2017)	NSE/BSE	Technical indicators	Exponential smoothing	LS-RF

Sharma & Juneja (2017) focus on making future stock market index value predictions using past data. The exploratory appraisal is based on 10-year historical data for two Indian equity markets, the CNX Nifty and the S&P BSE Sensex. Predictions are given for days 1 through 10, 15, 30, and 40 in the future. This study indicates utilizing LSboost to integrate the estimations and predictions from the ensemble of trees in a random forest. The suggested model’s prediction performance is contrasted with that of the well-known support vector regression. Each of the prediction models uses a different set of technical indicators as inputs.

## Challenges and discussion

Analysis and forecasting of the financial markets remain an intriguing and complex topic. Data is increasingly accessible, but it is becoming more challenging to gather and interpret in order to gain relevant insights and determine how it influences the investors in making their financial decisions wisely (Ozbayoglu, Gudelek & Sezer, 2020). It is challenging to extract characteristics from financial information as it is essential to address the many components used to generate forecasts. Financial datasets are frequently erratic and the data’s quality has a big impact on performing analysis and predictions.

Economic uncertainty is the degree of swings in an investment’s market price. Uncertainty and inflation are the key causes of volatility, and a volatile market increases the risk factor to greater extent. Also, volatility in trading constantly affects our emotions in negative ways that in turn makes the investors to take wrong decisions. When the marketplace is highly dynamic, predicting stock values becomes more complex. One such instance is the flash collapse, which erased $860 billion from US financial markets in the space of 30 min. The volatility of the stock market is also significantly influenced by international politics. So, it is difficult to ascertain the adequacy and effectiveness of these methods as new versions are constantly introduced into the market. This branch of study is exceptional in its self-defeating tendency. Simply speaking, sharing extremely successful techniques with other organizations will render such strategies redundant. Smartest algorithm trading is extremely restricted and confidential in the market segments. The technique or framework of such algorithms is never revealed.

Sentiment analysis using data derived from social media is receiving greater attention as a result of the growing influence of digital networking on many facets of our life (Yang et al., 2023). Due to multiple reasons, including misleading information and the bot data released on the web by different sources, this data can be volatile and challenging to interpret. Finding high-quality data and getting useful insights from it is difficult. Organizational quarterly or annual reports that are employed for stock prediction are a respectable choice or additional resource. These data, when correctly deciphered, provide important knowledge of an organization’s state, aiding in the comprehension of the stock’s future development. But in order to understand the fundamental details of the company requires professional qualification or knowledge. Since historical Twitter information cannot be obtained without someone saving it, data must be collected over a given time period starting with the given date and time, required data must be filtered out of the stream of irrelevant tweets, and validation is needed to access real-time Twitter data. The live testing of the forecast will again be a significant problem.

In order to understand the present literature status in the field of stock market analysis, an in-depth statistical survey was conducted, and the following section is focused on reviewing the research questions mentioned in the earlier section.

### RQ1: what are the research techniques or methodologies employed in recent times to predict the stock market movements?

**Figure 1:**
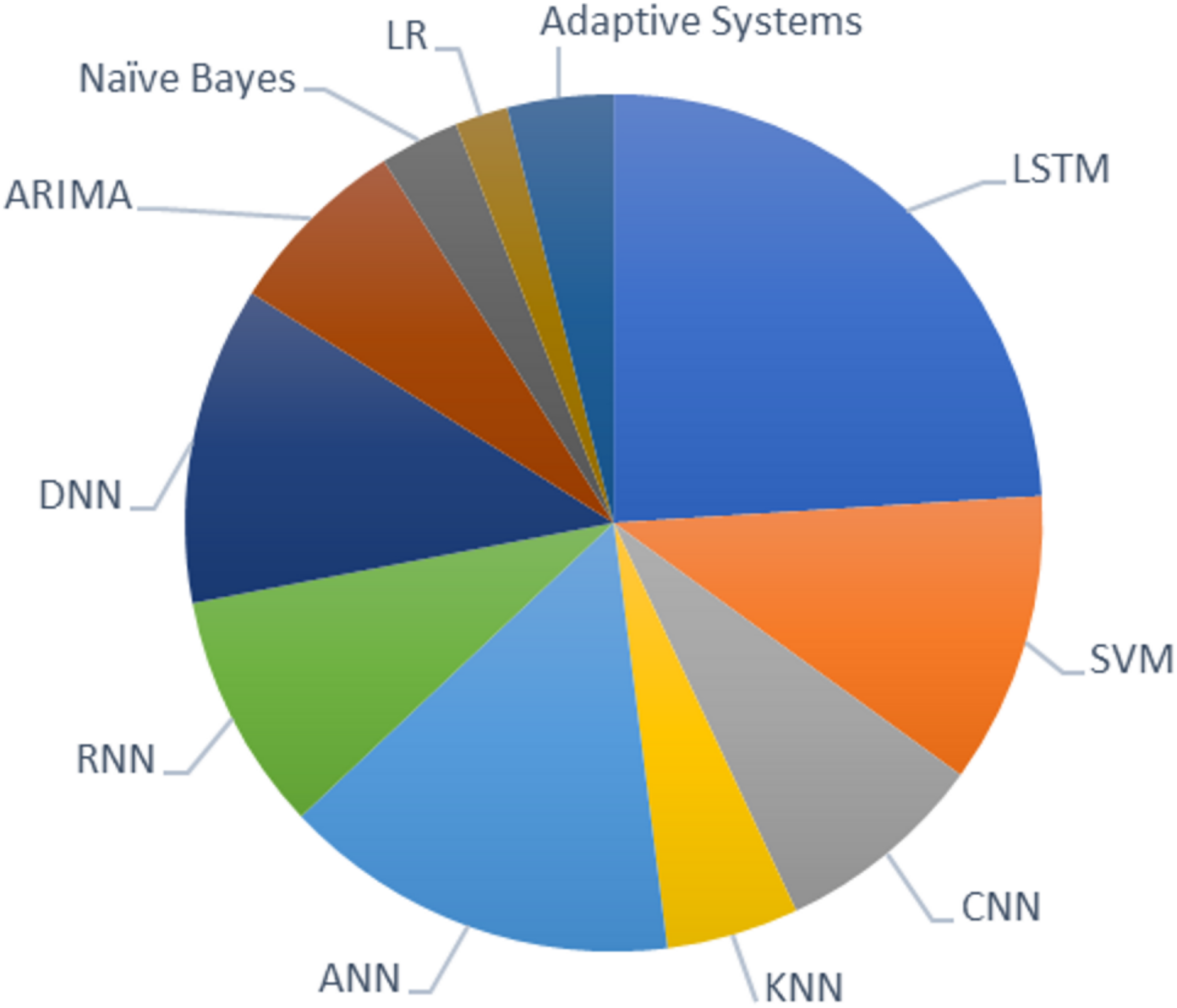
Recent techniques used in stock market prediction.

Based on the survey analysis, it is evident that LSTM is the most preferred model among researchers for predicting stock price movements. Other machine learning models like SVM, KNN, ANN, and CNN are widely used in many studies next to LSTM. Among researchers, CNN is mostly preferred algorithm for feature selection, and LSTM for stock predictions. Logistic regression and the adaptive system is the least preferred model for the chosen field of study. The graph in Fig. 1 shows the recent techniques used in the domain of stock market prediction.

### RQ2: what are the different sources of datasets considered for the stock market predictions?

**Figure 2:**
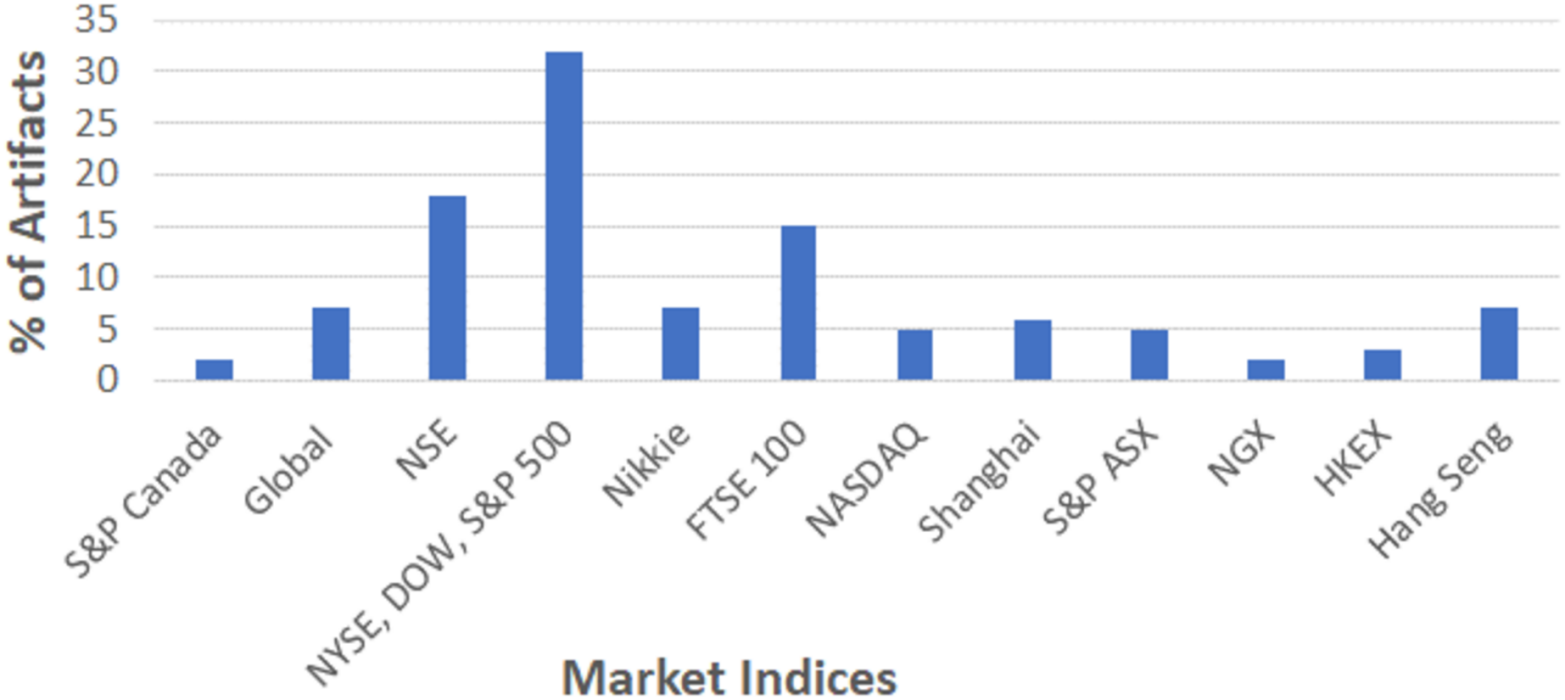
Sources of datasets used in stock market predictions.

As depicted in Fig. 2, some of the selected studies were considered generic datasets that are suitable for the global market. In general, the majority of analysts preferred US-based indices such as NASDAQ, Dow Jones, NYSE, and S&P 500 for their research analysis. Over 15% of studies were focused on Indian stock indices (NSE) followed by FTSE 100 (United Kingdom), Nikkei (Japan), Hang Sang (Hong Kong), Shangai (China), and others. For analysis, majority of studies used the dataset readily available in online platform like Kaggle, whereas few studies utilize the data directly from Yahoo finance.

### RQ3: what are the most popular journals available in the domain of stock market investments?

**Figure 3:**
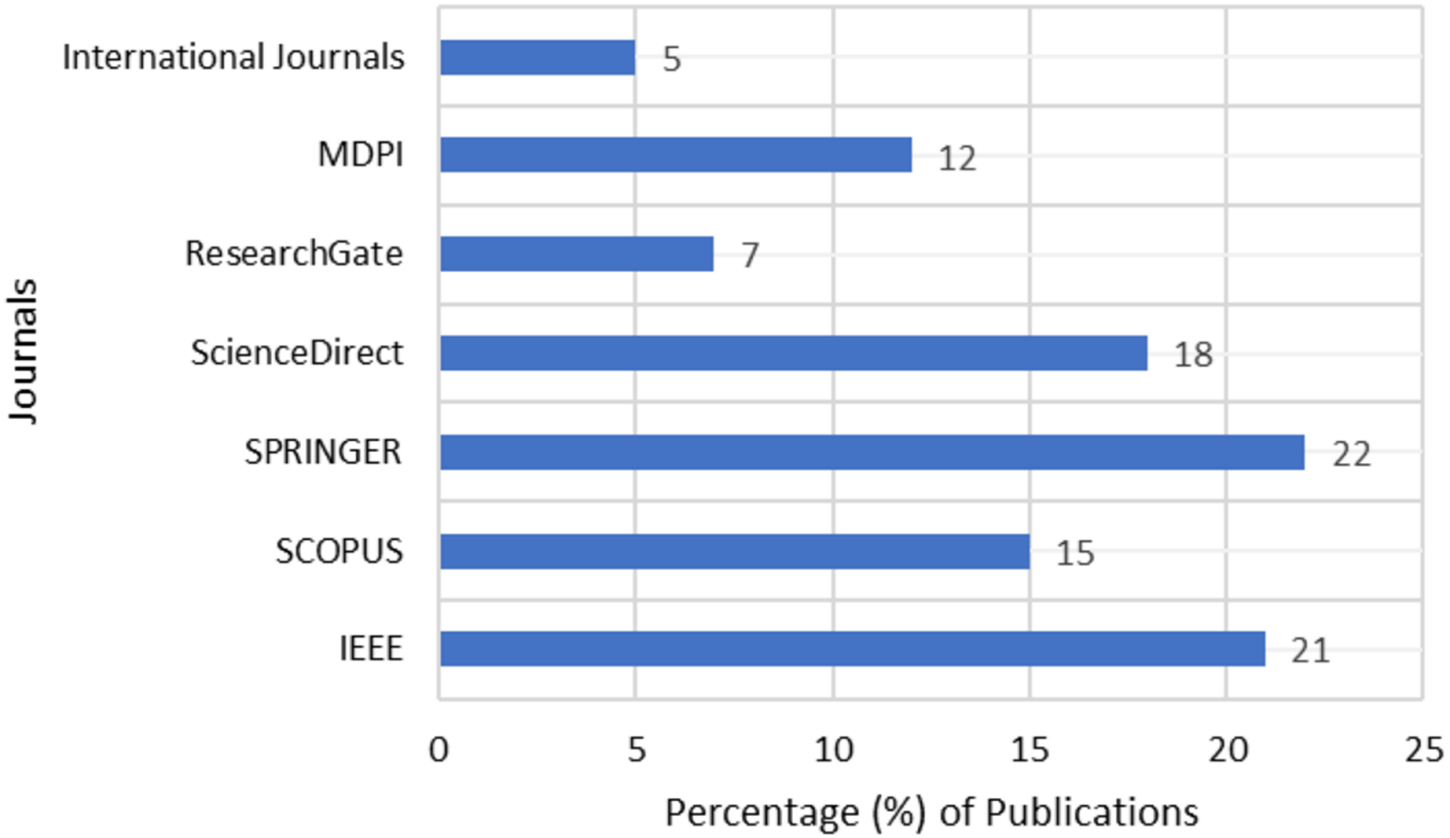
Publications of stock market analysis.

For this study, we have considered the research articles pertaining to stock market predictions from widely popular repositories like Springer, Elsevier SCOPUS, IEEE, and Science Direct. The data as represented in Fig. 3 shows sufficient research articles are available in the repositories to help the researchers and analysts with their research directions.

### RQ4: what are the countries that show research interests in equity/capital market investments?

**Figure 4:**
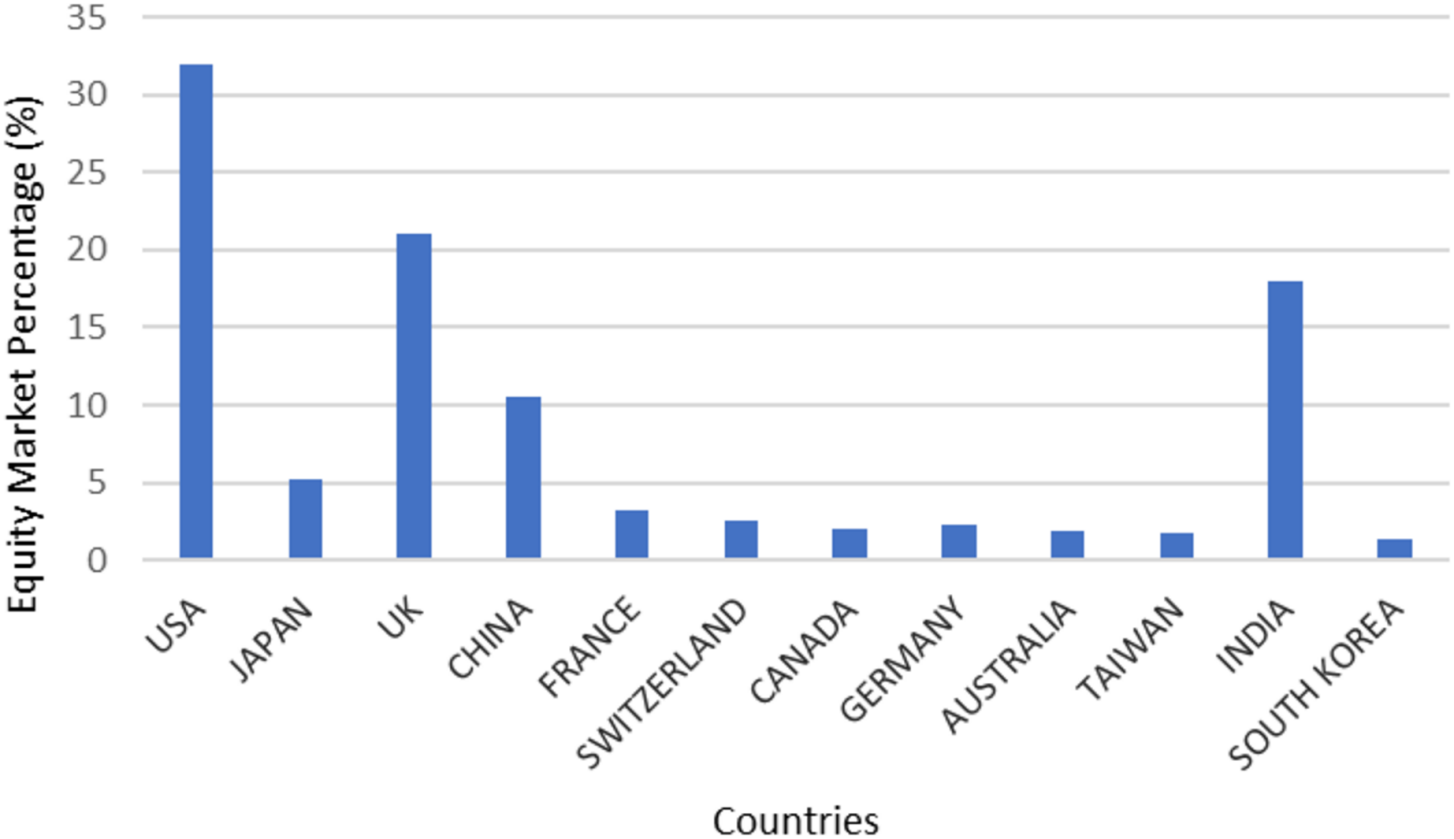
Countries interested in equity market.

According to this survey as represented in Fig. 4, the research works in the field of stock market analysis predominantly popular in four countries namely USA, UK, India, and China. This shows that the interests among participants are directly proportional to the growth potential of the country. The least amount of research work in this field was carried out by Japan, Canada, Australia, and other countries.

### RQ5: what are the most popular evaluation metrics used in a stock market analysis?

**Figure 5:**
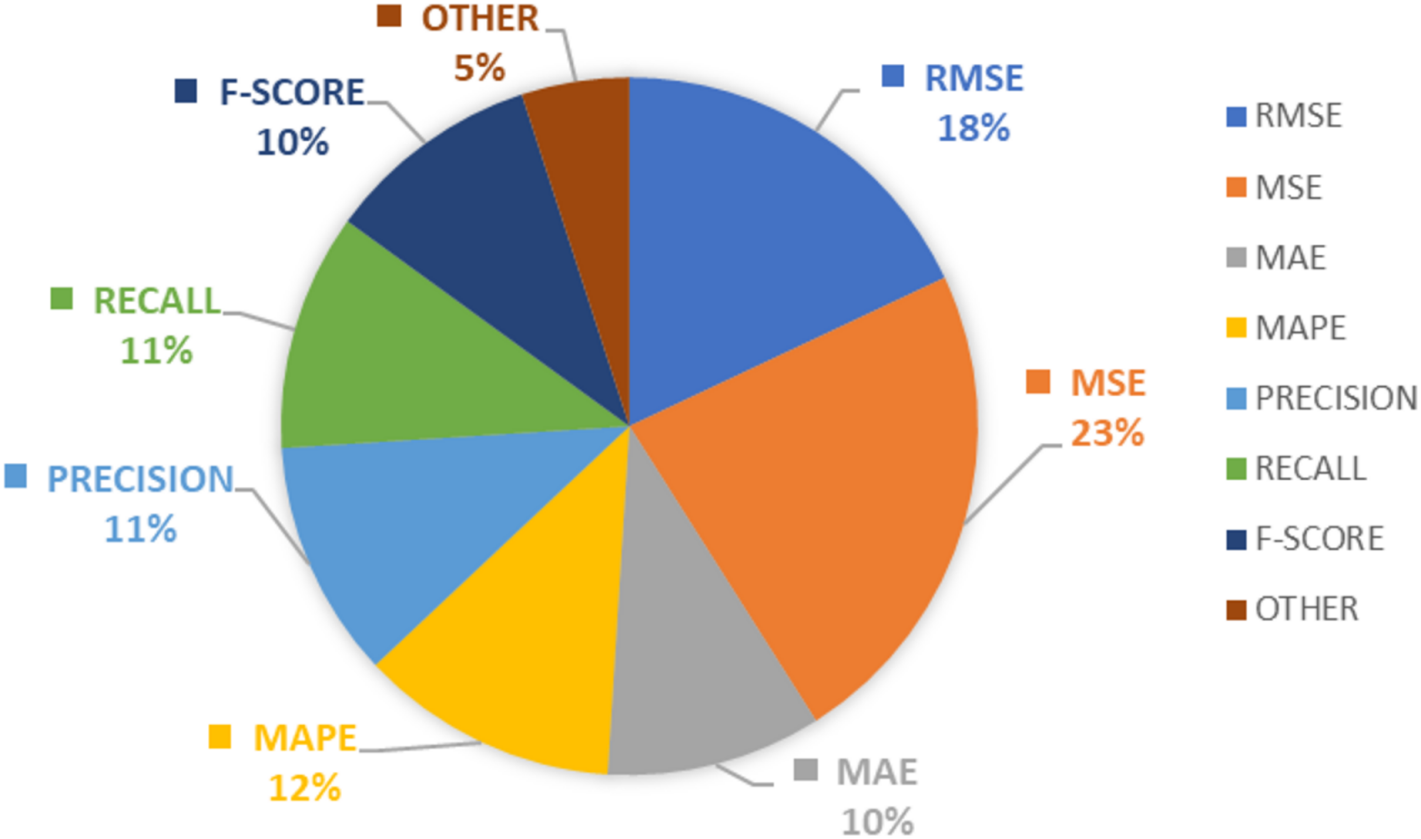
Evaluation metrics in stock market predictions.

According to this study, the finding shows MSE and RMSE considered as most popular evaluation metrics followed by accuracy, precession, recall, and F-Score. The majority of machine learning models used error metrics in combination with accuracy as the preferred evaluation metric, whereas deep learning techniques employed precision, recall, and F-score as the preferred choice. The details pertaining to the evaluation metrics in the domain of stock market analysis are denoted in Fig. 5.

## Conclusions

In this study, we have conducted a survey of over 100 research articles in the domain of stock market prediction utilizing recent machine learning approaches, neural networks, text analytics, and other approaches on various stock exchanges available globally. Due to the volatile nature of the financial markets, prediction plays a crucial part in the stock market company, which is a highly difficult and complex procedure. Based on the survey conducted, this study attempted to address the five key research questions about equity market investment areas. The main objective of this study is to support researchers, analysts, investors, and individual participants to take informed decisions in equity market financing.

## Future scope

Other influences and aspects, such as financial ratios, numerous cases, *etc*., should be included in the future scope. As additional variables are incorporated, the performance will improve. The procedures could also be used to analyze the substance of comments on social media in order to find trends or links between clients and firm representatives. The overall performance structure of the firm may also be predicted with the use of conventional algorithms and data mining approaches. Future studies might focus on combining data from stock sentiment categorization with quantitative numbers pertaining to prior stock values to anticipate financial markets. More effective stock assessment systems may be constructed by combining both types of information. Deep learning-based strategies may be used to improve the effectiveness of feature extraction techniques. Graph knowledge approaches are a potential technology for developing the performance of the proposed engines; nevertheless, future studies ought to concentrate on the complexity and gradient of networking with plenty of nodes. There is potential for predicting stock market patterns during pandemics utilizing neural network algorithms, such as the LSTM and GRU (Gated Recurrent Units) techniques, which have been proven to be significantly successful in time series data prediction applications.

The complete versions of all the acronyms discussed in our study are presented in Table 5.

**Table 5:** Acronyms used in this article.

Abbreviation	Full form
KSE	Karachi stock exchange
NSE	National stock exchange of India
NYSE	New York stock exchange
LSE	London stock exchange
NASDAQ	National association of securities dealers automated quotations
SSE	Shanghai stock exchange
GSE	German stock exchange
TSE	Tokyo stock exchange
ISE	Istanbul stock exchange
CSE	Colombo stock exchange
NGX	Nigerian exchange
MOEX	Moscow exchange
B3	Brazil stock exchange
HKEX	Hong kong exchanges
SLP	Single layer perceptron
MLP	Multi layer perceptron
RBF	Radial basis function
SVM	Support vector machine
PR	Polynomial regression
LR	Linear regression
RF	Random forest
KNN	K-nearest neighbor
RNN	Recurrent neural network
LSTM	Long short-term memory
CNN	Convolutional neural netwotk
ANN	Artificial neural network
ET	Extra tree
GBM	Gradient boosting classifier
ARIMA	Auto-regressive integrated moving average
3MMA	Three month moving average
SGD	Stochastic gradient descent
DT	Decision tree
ES	Exponential smoothing
ExtRa	Extremely randomized trees
PLS	Partial least square
NB	Naïve Bayes
SMO	Sequential minimal optimization
GAM	Generalized additive model
GA	Generic algorithm
FA	Fuzzy algorithms
DNN	Dense neural network
RA	Regression algorithms
HA	Hybrid approaches
SOM	Self-organising maps
SVR	Support vector regression
MCS	Monte Carlo simulation
CART	Classification and regression trees
GP	Gaussian processes
BSM	Black Scholes model
GRNN	Generalized regression neural network
BPNN	Back propagation neural network
HMM	Hidden Markov model
EMD	Empirical mode decomposition
ANFIS	Adaptive neuro-fuzzy inference system
GGAP	General growing and pruning
CSWNN	Cuckoo search wavelet neural network
WNN	Wavelet neural network
NISI	New investor sentiment index
SENN	Stockensemble-based neural network
GC-CNN	Graph convolutional neural network
AD-GAT	Attribute-driven graph attention network
AA	Auditory algorithm
FNN	Feed forward neural network
SDE	Stochastic differential equations
GBM	Geometric Brownian motion
LS-FR	Random forest using LSBoost

## Additional Information and Declarations

### Data Availability

The following information was supplied regarding data availability: This is a survey article and hence did not utilize code/data.
